# Power Doppler Ultrasound Assessment of A1 Pulley. A New Target of Inflammation in Psoriatic Arthritis?

**DOI:** 10.3389/fmed.2020.00204

**Published:** 2020-06-05

**Authors:** Gianluca Smerilli, Edoardo Cipolletta, Marco Di Carlo, Andrea Di Matteo, Walter Grassi, Emilio Filippucci

**Affiliations:** ^1^Rheumatology Unit, Department of Clinical and Molecular Sciences, Polytechnic University of Marche, “Carlo Urbani” Hospital, Jesi, Ancona, Italy; ^2^Leeds Institute of Rheumatic and Musculoskeletal Medicine, University of Leeds, Leeds, United Kingdom

**Keywords:** ultrasonography, psoriatic arthritis, rheumatoid arthritis, diagnostic imaging, annular pulley

## Abstract

**Objective:** To determine the prevalence of grey scale and power Doppler (PD) ultrasound (US) features of A1 pulley inflammation in a cohort of psoriatic arthritis (PsA) patients compared with rheumatoid arthritis (RA) patients.

**Methods:** Sixty patients (30 with PsA and 30 with RA) were consecutively enrolled. The main clinimetric indexes were recorded, and US assessment of A1 pulleys from second to fifth fingers bilaterally was carried out. The presence of A1 pulley inflammation, defined as PD signal within a thickened pulley, was registered.

**Results:** A1 pulley inflammation was found in 15 of 240 fingers (6.3%) of eight PsA patients (26.7%) and in one of 240 fingers (0.4%) of one RA patient (3.3%) (*p* < 0.01 and *p* = 0.03, respectively). Seven of eight PsA patients (88%) with at least one inflamed A1 pulley had a moderate/high disease activity score. The regression linear analysis (*R*^2^ = 0.36, adjusted *R*^2^ = 0.31) showed that A1 pulley inflammation was correlated with Disease Activity Index for Psoriatic Arthritis (DAPSA) (β = 0.43, *p* = 0.03).

**Conclusion:** US A1 pulley inflammation appears to be relatively common at patient level in PsA, seems to be a characteristic feature of PsA compared to RA, and correlates with DAPSA.

## Introduction

Finger flexor tendons and their surrounding synovial sheaths are located in osteofibrous channels, which are composed by the palmar aspect of the phalanges and metacarpal heads and by the digital fibrous tendon sheaths made by the digital pulleys (1). These structures are divided into annular pulleys (A1–A5) and cruciform pulleys, and their main function is to stabilize the tendons during finger flexion (1).

While feasibility of high-frequency ultrasound (US) assessment of annular pulleys has been well documented (2, 3), cruciform pulleys are not easily depictable with US because of their small size (3).

A1 pulley thickening has been recognized having a key role in the pathogenesis of “trigger finger,” and US has proven to be useful in depicting this morphostructural abnormality (4).

In 2015, a magnetic resonance imaging (MRI) study turned the attention to the annular pulleys, demonstrating that these are common targets of inflammation in psoriatic arthritis (PsA) patients with dactylitis (5). Since then, two US studies evaluated the thickness of annular pulleys in chronic arthropathies, and both concluded that this is increased in PsA patients compared to rheumatoid arthritis (RA) patients and healthy subjects (6, 7). A possible link between A1 pulley thickening and dactylitis through a “deep Koebner” phenomenon was hypothesized because of the correlation between this finding and previous dactylitis (7). In a case report, inflammatory involvement of annular pulleys was depicted by US in a PsA patient (8), and power Doppler (PD) enhancement of annular pulleys was found in active psoriatic dactylitis (9). Moreover, in a very recent article, MRI signs of inflammatory involvement of annular pulleys were more often encountered in a small cohort of PsA patients compared to RA patients and healthy controls (10).

Thus, we believe that PD US potential in the assessment of A1 pulley involvement in PsA has not been adequately investigated yet.

The main aim of the present study was to determine the prevalence of PD US findings indicative of A1 pulley inflammation in PsA patients and in controls with RA.

## Materials and Methods

### Patients

Consecutive patients with PsA according to the Classification Criteria for Psoriatic Arthritis criteria (11) and controls with RA fulfilling the American College of Rheumatology/European League Against Rheumatism (ACR/EULAR) criteria (12) were enrolled at the Rheumatology Unit of “Carlo Urbani” Hospital, in Jesi (Ancona, Italy). Patients younger than 18 years were excluded.

The study was conducted in accordance with the Helsinki Declaration and was approved by the local ethics committee. All patients signed informed consent.

### Clinical Examination

A rheumatologist (E.C.) recorded for each patient the presence of “trigger finger”, current or previous dactylitis (second to fifth fingers bilaterally), and tenderness in the A1 pulley region (second to fifth fingers bilaterally). The main clinimetric indexes (Disease Activity Index for PSoriatic Arthritis [DAPSA] in PsA patients and 28-joint Disease Activity Score [DAS28-C reactive protein (CRP)] in RA patients) were calculated.

Disease activity was interpreted according to the following cutoff values: DAPSA >28 and DAS28 >5.1 correspond to high disease activity, 28 ≥ DAPSA > 14 and 5.1 ≥ DAS28 ≥ 3.2 to moderate disease activity, 14 ≥ DAPSA > 4 and 3.2 > DAS28 ≥ 2.6 to low disease activity, and DAPSA ≤ 4 and DAS28 <2.6 to a remission status.

### US Assessment

On the same day, another rheumatologist (G.S.) blinded to clinical data performed all the US examinations using a MyLabClassC (Esaote, Genova, Italy) equipped with a 10- to 22-MHz linear transducer. Patients were asked not to talk about their clinical condition with the sonographer.

**Figure 1:**
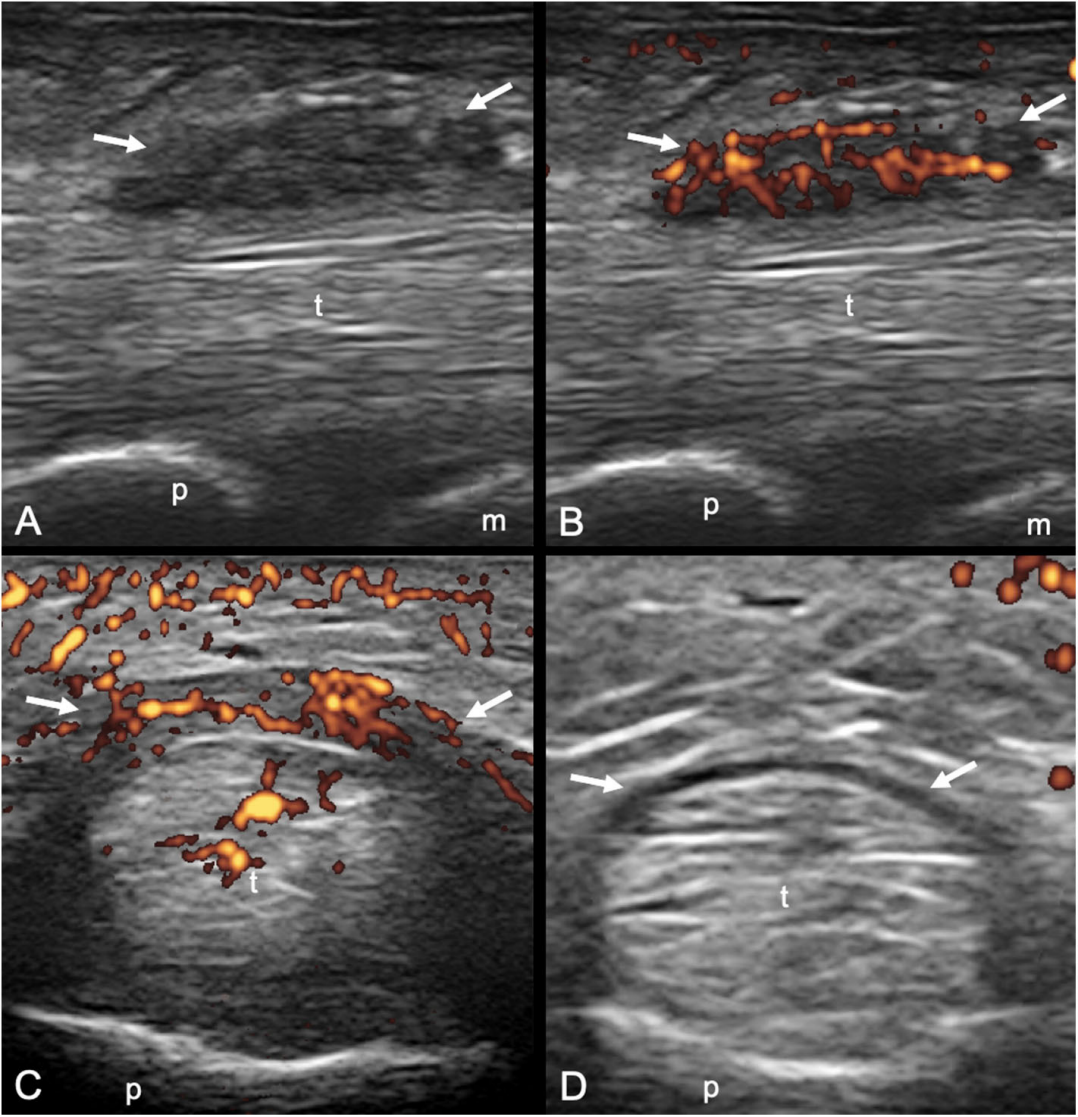
Psoriatic arthritis. Longitudinal **(A,B)** and transverse **(C,D)** scans of the annular pulley A1 using a 22-MHz probe. In **(A,B)** a markedly thickened and inflamed A1 pulley (arrows) is depicted without and with power Doppler mode, respectively. In **(C,D)** a right–left comparison of the A1 pulley (arrows) of the third finger of another patient is illustrated. Note the thickening of the inflamed pulley and the presence of power Doppler within it in **(C)** compared to **(D)**. In **(C)** concomitant intratendinous power Doppler signal can be appreciated. m, metacarpal head; p, proximal phalanx; t, finger flexor tendons.

**Figure 2:**
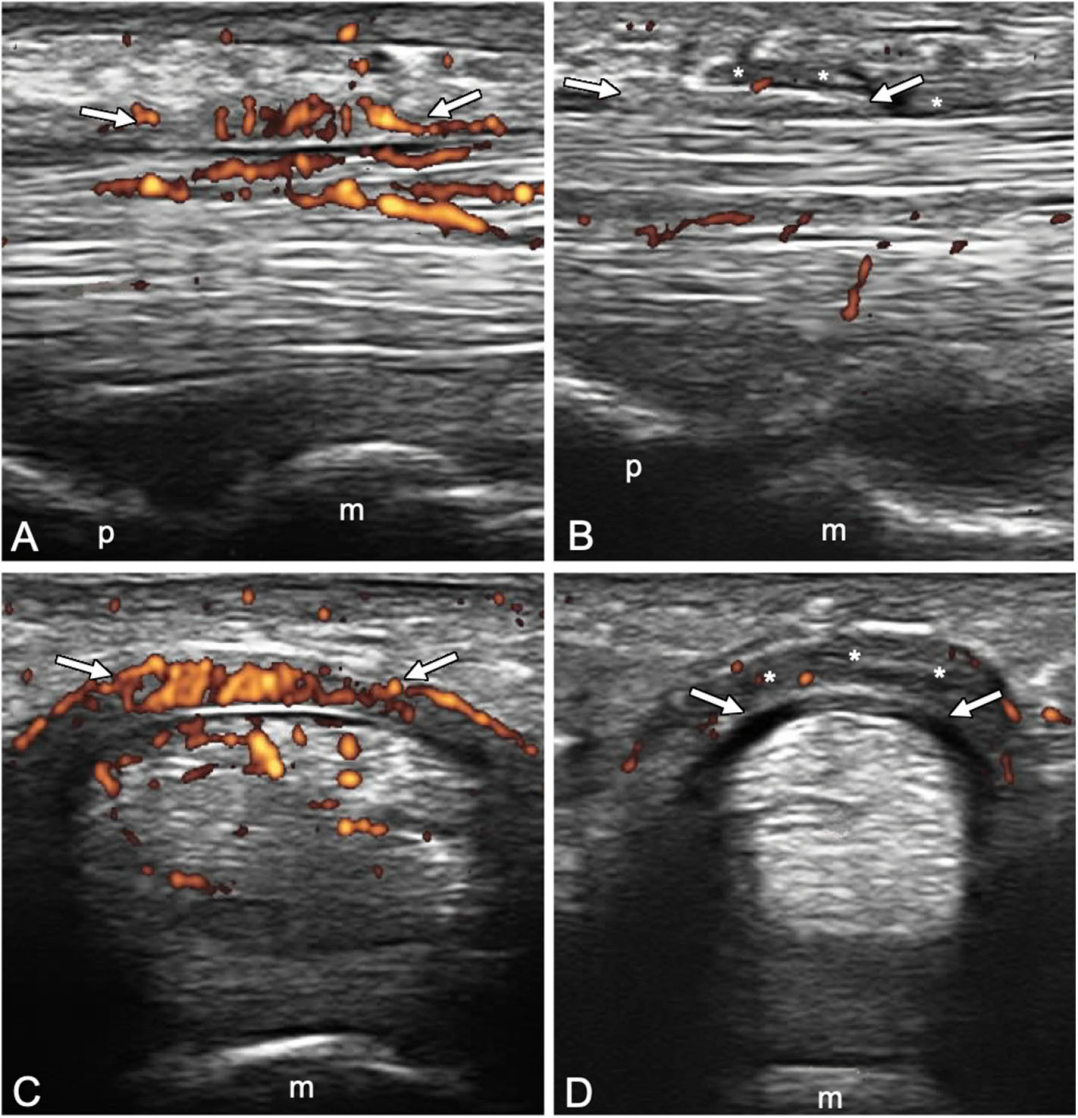
Longitudinal **(A,B)** and transverse **(C,D)** scans of the annular pulley A1 using a 22-MHz probe in psoriatic arthritis **(A,C)** and rheumatoid arthritis **(B,D)** patients. In **(A,C)**, A1 pulley (arrows) inflammation is depicted (power Doppler signal inside a thickened pulley). Power Doppler signal is also noticeable within the superficial flexor tendon in the portion closest to the pulley. In **(B,D)**, A1 pulley (arrows) is not inflamed. The presence of synovial proliferation in the tendon sheath spilling over the normal pulley is shown (asterisks), representing a potential diagnostic pitfall. m, metacarpal head; p, proximal phalanx.

A1 pulley from second to fifth fingers were assessed bilaterally adopting longitudinal and transverse scans as indicated by the 2017 EULAR standardized procedures for US imaging in rheumatology (13). The following pathological US findings were recorded: inflammation of the pulley (defined as the presence of PD signal within a thickened pulley) and tenosynovitis of the digital flexor tendons at finger level according to OMERACT definition (14). Thickening of the pulley was defined comparing the assessed structure with the adjacent and contralateral fingers (2). Particular attention was paid to the correct distinction between inflammatory involvement of the A1 pulley and tenosynovitis of flexor tendons (Figures 1, 2). A thorough US examination with dynamic assessment during passive finger flexion and extension was conducted to distinguish the pulley, which stays still, from the tendons and the tenosynovial proliferation, which move with the finger.

### Statistical Analysis

The association between the sonographic findings and clinical–demographic data was tested using Cramer's *V* (*V*), point-biserial correlation (Rpb), and Spearman correlation coefficient (*R*). Stepwise linear regression analysis was performed to define predictive values of A1 pulley inflammation. DAPSA was used as the dependent variable, whereas independent variables were the presence of pulley inflammation and the presence of tenosynovitis. Statistical analysis was performed using SPSS (Chicago, IL, USA).

## Results

Sixty patients were enrolled in this cross-sectional and monocentric study: 30 with PsA and 30 with RA. Table 1 reports the demographic and clinical characteristics of PsA and RA patients.

**Table 1:** Demographic and clinical characteristics of PsA and RA patients.

	**PsA (*n* = 30)**	**RA (*n* = 30)**
Age, mean ± SD (years)	58.6 ± 10.8	58.5 ± 13.6
Female/male	14/16	23/7
BMI, mean ± SD (kg/m^2^)	25.9 ± 2.9	25.0 ± 5.4
R/L dominant hand	27/3	30/0
Trigger fingers (%)	2 (6.7)	2 (6.7)
Previous hand dactylitis (%)	8 (26.6)	0 (0)
Current hand dactylitis (%)	1 (3.3)	0 (0)
**WORKING CONDITION**		
Blue collar workers (%)	16 (53.3)	7 (23.3)
White collar workers (%)	5 (16.7)	5 (16.7)
Retired/unoccupied (%)	9 (30.0)	18 (60.0)
**DISEASE ACTIVITY**		
Disease duration, mean ± SD (years)	7.5 ± 10.1	6.0 ± 7.3
Disease activity (DAPSA/DAS28), mean ± SD	14.2 ± 11.2	3.4 ± 1.3
Remission/low disease activity	16 (53.3)	16 (53.3)
Moderate/high disease activity	14 (46.7)	14 (46.7)
**TREATMENT**		
cDMARDs (%)	13 (43.3)	17 (56.7)
bDMARDs (%)	17 (56.7)	8 (26.7)
Steroid (%)	2 (6.7)	13 (43.3)

*bDMARDs, biologic disease-modifying antirheumatic drugs; BMI, body mass index; cDMARDs, conventional disease-modifying antirheumatic drugs; DAPSA, Disease Activity in PSoriatic Arthritis score; DAS28, Disease Activity Score in 28 joints for rheumatoid arthritis; L, left; PsA, psoriatic arthritis; R, right; RA, rheumatoid arthritis; SD, standard deviation*.

Tenderness of the volar side of the metacarpophalangeal joint was reported in 28 fingers (11.7%) of 13 PsA patients (43.3%) and in 13 fingers (4.6%) of six RA patients (20.0%).

### US Findings

Inflammation of A1 pulley was found in 15 of 240 fingers (6.3%) of eight PsA patients (26.7%) and in one of 240 fingers (0.4%) of one RA patient (3.3%) (*p* < 0.01 and *p* = 0.03, respectively). Inflammation of A1 pulley in the absence of tenosynovitis was reported in six A1 pulleys (2.5%) of six PsA patients (20.0%).

No significant difference was reported regarding the prevalence of flexor finger flexor tendons between PsA [22 fingers (9.2%) in 10 PsA patients (33.3%)] and RA patients [21 fingers (8.8%) in six RA patients (20.0%)] (*p* = 1.0 and *p* = 0.24, respectively).

### Correlation Between Demographic, Clinical, and Sonographic Data

Both pulley inflammation and tenosynovitis were correlated with DAPSA (Rpb = 0.56, *p* < 0.01, and Rpb = 0.48, *p* < 0.01). In fact, seven of eight PsA patients (88%) with at least one inflamed A1 pulley had a moderate/high disease activity score.

The regression linear analysis (*R*^2^ = 0.36, adjusted *R*^2^ = 0.31) showed that A1 pulley inflammation was predictive of higher DAPSA scores (β = 0.43, *p* = 0.03), whereas tenosynovitis did not reach statistical significance (β = 0.25, *p* = 0.18).

Both A1 pulley inflammation and flexor tendons tenosynovitis were associated with tenderness (*V* = 0.55, *p* < 0.01, and *V* = 0.38, *p* < 0.01).

No significant association was reported between A1 pulley inflammation and past or current episodes of dactylitis (*p* = 0.09). However, the only current dactylitis assessed showed A1 pulley inflammation. No significant correlations were found between A1 pulley inflammation and other demographic variables.

No significant association was found between the presence of at least one inflamed A1 pulley and working condition (*p* = 0.84).

## Discussion

The identification of the pathophysiological processes underlying the inflammatory involvement of periarticular structures during PsA is still a fascinating area of research, and US is capable of providing a clear depiction of finger pathology in PsA (15–19).

Annular pulleys are functional entheses, being subjected to very high shear stress provoked by continuous friction with adjacent tendons (7). The repetitive microtrauma makes them an ideal target for PsA inflammation, which can often be triggered mechanically. On the other hand, RA has a predilection for synovial structures; thus, we hypothesized that A1 pulley could be relatively spared.

To the best of our knowledge, this is the first study that compared PD US findings indicative of inflammation at A1 pulley level in PsA and RA patients. As expected, inflammation of A1 pulley was relatively common in PsA patients, whereas it was found in only one of 240 RA fingers examined. Moreover, the only inflamed A1 pulley in RA was found in a clinically diagnosed trigger finger and may therefore not be a RA manifestation in that patient. Indeed, an increased PD signal within the pulley has been described in trigger finger (4). However, according to our preliminary data, A1 pulley inflammation is only rarely associated with trigger finger symptoms in PsA [one of 15 (6.6%)].

This finding, in our PsA cohort, was correlated with a higher disease activity (DAPSA), and this is an interesting aspect to be explored in future research.

The link between pulleys involvement and psoriatic dactylitis is currently a topic of growing interest. In fact, pulleys are thicker in fingers previously affected by dactylitis, and recently PD signal within pulleys has been found to be common in dactylitic fingers, with a prevalence reaching 51% for A1 pulley (7, 9). The absence of a clear correlation in our cohort between A1 pulley inflammation and previous dactylitis raises an interesting question about the chronological relationship of pulley inflammation with dactylitis, and a prospective study may shed light on this possibly crucial pathogenetic moment.

The main limitations of our study were the small number of patients assessed and the fact that we only assessed A1 pulley. Another limitation of our study was that the two groups were not matched for working condition. However, no significant association was found between the presence of A1 pulley inflammation and working condition at patient level, and five of nine patients (55.5%) with at least one A1 pulley inflamed were not blue collar workers.

One of the limitations acknowledged by the authors in a recent article (9) was that the presence digital flexor tendons tenosynovitis could be misinterpreted as pulley inflammation. However, high-frequency US probes allow an excellent anatomical resolution of small structures such as A1 pulley, and a detailed and dynamic US examination with longitudinal and transverse scans allows distinguishing tenosynovitis from inflammation of the pulley with great accuracy, avoiding this potential pitfall.

To summarize, in the present pilot study, we found that A1 pulley inflammatory involvement is not uncommon in PsA at patient level, seems to be a characteristic feature of PsA compared to RA, and correlates with disease activity.

## Data Availability Statement

The datasets generated for this study are available on request to the corresponding author.

## Ethics Statement

The studies involving human participants were reviewed and approved by CERM MARCHE. The patients/participants provided their written informed consent to participate in this study.

## Author Contributions

GS, EC, and EF substantially contributed to study conception and design, acquisition of data, and analysis and interpretation of data. MD substantially contributed to acquisition of data, and analysis and interpretation of data. WG and AD substantially contributed to study conception and design. All the authors revised the paper and approved the final version of the article to be published.

## Conflict of Interest

EF has received speaking fees from AbbVie, Bristol -Myers Squibb, Novartis, Pfizer, Roche, and Union Chimique Belge Pharma. WG has received speaking fees from AbbVie, Celgene, Grünenthal, Pfizer, and Union Chimique Belge Pharma. This study was conducted while AD was an ARTICULUM fellow. The remaining authors declare that the research was conducted in the absence of any commercial or financial relationships that could be construed as a potential conflict of interest.
